# Anorexia Nervosa With Intermittent Fever Due to Diet-Induced Thermogenesis: A Case Report

**DOI:** 10.7759/cureus.53647

**Published:** 2024-02-05

**Authors:** Atsuhiro Ijiri, Soichiro Seno, Nobuaki Kiriu, Hiroshi Kato, Tetsuro Kiyozumi

**Affiliations:** 1 Department of Traumatology and Critical Care Medicine, National Defense Medical College, Tokorozawa, JPN

**Keywords:** high protein, fever, tube feeding, anorexia nervosa (an), nutrition and metabolism, diet-induced thermogenesis

## Abstract

Diet-induced thermogenesis, influenced primarily by protein intake, generates energy from food. Herein, we present the case of anorexia nervosa in a 30-year-old woman, who developed intermittent fever while transitioning from continuous to intermittent tube feeding, with an increase in protein intake. Extensive investigations ruled out infection- or drug-related causes, indicating that intermittent fever resulted from diet-induced thermogenesis due to high protein administration. Recognizing the potential for diet-induced thermogenesis in cases of fever during tube feeding is crucial to avoid unnecessary antibiotic use and prevent the discontinuation of essential medications.

## Introduction

Diet-induced thermogenesis (DIT) refers to the energy generated and consumed during eating [1], constituting approximately 10% of daily energy consumption [2]. Anorexia nervosa is characterized by distorted perceptions of weight or body shape, excessive influence of body image on self-evaluation, or a persistent lack of awareness of the current underweight status [3]. Patients with anorexia nervosa often exhibit hypermetabolism, leading to higher energy expenditure through DIT compared to that in healthy individuals [4]. This report presents a case of anorexia nervosa treated with high-protein tube feeding, resulting in intermittent fever attributed to DIT. To our knowledge, this is the first case report of DIT-induced fever in a patient with anorexia nervosa.

## Case presentation

A 30-year-old woman was diagnosed with schizophrenia four months previously owing to auditory hallucinations and delusional symptoms. Additionally, she had been experiencing anorexia for a week after the initial diagnosis. Over four days, she encountered difficulty in walking, prompting her to seek medical attention from her previous doctor. Eventually, she was admitted to our emergency department with suspected anorexia nervosa and refeeding syndrome. On admission, the patient was 152 cm tall and weighed 20.1 kg (body mass index (BMI), 8.7 kg/m^2^). Her vital signs were as follows: neurological scoring system, E3V4M6; blood pressure, 120/88 mmHg; heart rate, 126 beats/minute; respiratory rate, 13 breaths/minute; 100% oxygen saturation (room air), body temperature, 35.7°C; and pupils, 4 mm/4 mm with bilateral rapid light reflex. General and systemic examinations were unremarkable, except for the markedly thin body. Laboratory tests revealed elevated liver function test, dehydration, and malnutrition (Table 1).

**Table 1:** Laboratory findings at admission to the ED. ED: emergency department; WBC: white blood cell count; RBC: red blood cell count; Hb: hemoglobin; Plt: platelet count; APTT: activated partial thromboplastin time; PT-INR: prothrombin time-international normalized ratio; Fbg: fibrinogen; T-Bil: total bilirubin; D-Bil: direct bilirubin; TP: total protein; ALB: albumin; AST: aspartate aminotransferase; ALT: alanine aminotransferase; γ-GTP: gamma-glutamyl transferase; BUN: blood urea nitrogen; Cre: creatinine; CCr: creatinine clearance; AMY: amylase; LDH: lactate dehydrogenase; CK: creatine kinase; GLU: glucose; CRP: C-reactive protein; TSH: thyroid-stimulating hormone; FT4: free thyroxine; Tf: transferrin; TTR: transthyretin; RBP: retinol-binding protein

Variables	Results	Normal range
Complete blood cell count		
WBC (10^3^/µL)	11	3.3–8.6
RBC (10^6^/µL)	5.15	3.86–4.92
Hb (g/dL)	15.7	11.6–14.8
Plt (10^3^/µL)	254	158–348
Blood coagulation		
APTT (second)	31	24.0–32.0
PT-INR	1.1	0.90–1.10
Fbg (mg/dL)	217	180–400
D-dimer (µg/mL)	<0.5	<1.0
Biochemical test		
T-Bil (mg/dL)	2.51	0.2–1.2
D-Bil (mg/dL)	0.6	0.1–0.4
TP (g/dL)	5.9	6.5–8.2
ALB (g/dL)	3.5	3.8–5.2
AST (U/L)	405	8.0–30
ALT (U/L)	434	5.0–35
γ-GTP (U/L)	165	7.0–40
BUN (mg/dL)	66	8.0–20
Cre (mg/dL)	0.55	0.44–0.78
CCr (mL/minute)	47.4	70–130
AMY (U/L)	221	29–132
LDH (U/L)	307	100–225
CK (U/L)	49	<160
GLU (mg/dL)	70	65–110
Na (mmol/L)	146	135–147
K (mmol/L)	4	3.5–5.0
Cl (mmol/L)	109	98–108
Ca (mg/dL)	8.9	8.5–10.3
IP (mg/dL)	3.9	2.3–4.5
Zn (µg/dL)	63	80–130
CRP (mg/dL)	<0.3	<0.3
Endocrine test		
TSH ((µIU/mL)	2.61	0.61–4.23
FT4 (ng/mL)	1.05	0.68–1.26
Immunological test		
Tf (mg/dL)	106	190–320
TTR (mg/dL)	9.5	22–40
RBP (mg/dL)	1.4	1.9–4.6

**Figure 1:**
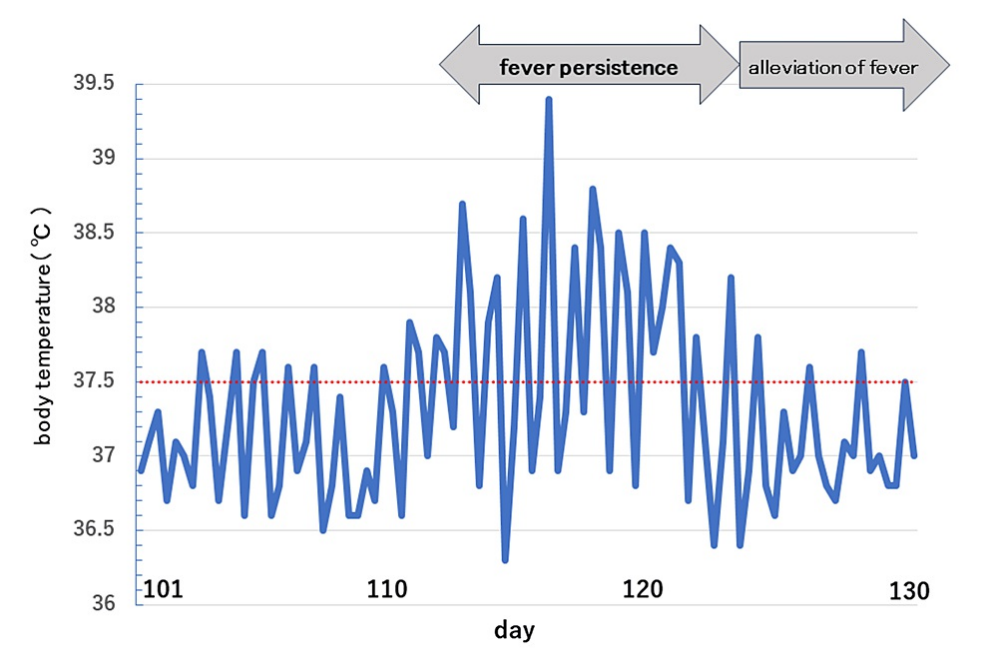
Temperature chart. The patient experienced fever starting from day 113 and it persisted until day 123.

**Figure 2:**
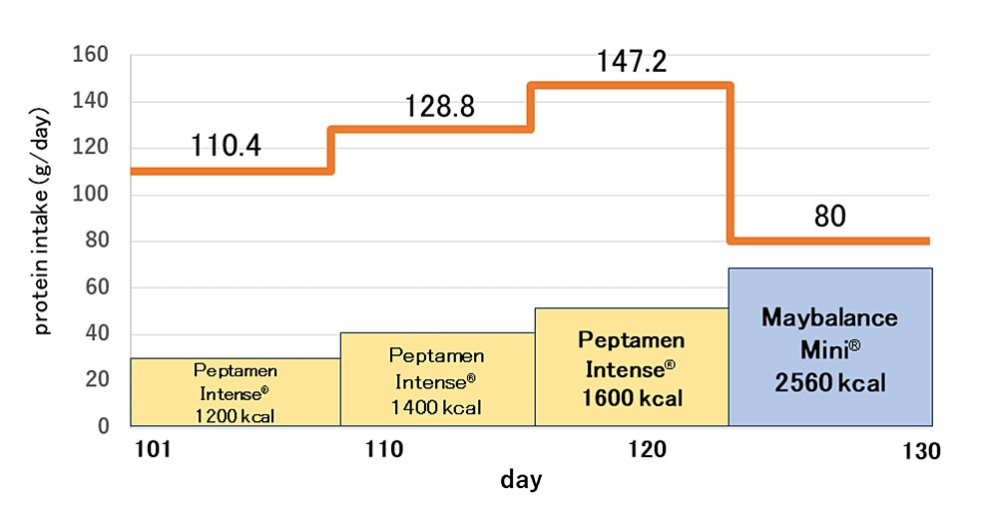
Tube feeding intake. The dose of Peptamen Intense® was increased on days 109 and 116 and switched to Maybalance Mini® on day 123.

Owing to the risk of refeeding syndrome, she was admitted for acute nutritional management. The management approach involved a gradual increase in the intake of Maybalance Mini® (Meiji Co., Ltd., Tokyo, Japan; 160 kcal, 5 g of protein, 25.4 g of carbohydrates/100 mL) in a stepwise manner. The dose was initiated at 10 mL/hour continuously and increased by 10 mL/hour every two days. If vomiting or bloody stools occurred during the treatment, the dosage was discontinued or reduced as deemed appropriate. Although there was a temporary weight gain up to 24.9 kg and normalization in liver function test results compared to the blood test results one week earlier, the aspartate aminotransferase, alanine transaminase, and gamma-glutamyl transferase (γ-GTP) levels increased from 55 to 233 U/L, 76 to 334 U/L, from 35 to 99 U/L, respectively, upon increasing the carbohydrate content in tube feeding (54 g of protein/day, 1,728 kcal/day). Abdominal computed tomography and ultrasound showed no mass lesions or morphologic abnormalities in the liver. Subsequently, the tube feeding method was changed to continuous administration of Peptamen Intense® (Nestle, Vevey, Switzerland; 100 kcal, 9.2 g of protein /100 mL) (110.4 g of protein /day, 1,200 kcal/day). On day 109, the patient’s weight reached 21.9 kg (BMI, 9.1 kg/m^2^); therefore, the tube feedings were changed from continuous to intermittent, and the protein intake was increased (128.8 g of protein/day, 1,400 kcal/day). Although the patient developed a fever on day 113, the feeding dose increased (147.2 g of protein/day, 1,600 kcal/day) as the patient’s weight had decreased to 21.2 kg on day 116. This change caused an intermittent fever that persisted for 11 days from day 113 to day 123 (Figures 1, 2).

During this period, body temperatures peaked at 39.4°C, the heart rate was 140 beats/minute, and the respiration rate was 22 breaths/minute. In addition to a good general condition and the absence of subjective symptoms, extensive culture tests (blood, urine, and sputum) revealed no causative organisms, and the C-reactive protein level was below the sensitivity threshold. Imaging studies were performed to check for any new infections, and no new medications were administered. The fever was diagnosed as DIT; therefore, the patient was switched back to Maybalance Mini® with reduced protein intake (96 g of protein/day, 2,560 kcal/day). The fever resolved on day 124 (the day after tube feeding was changed from Peptamen Intense® to Maybalance Mini®). Her weight improved to 38.4 kg (BMI, 16.7 kg/m^2^) on day 284, and she started eating solid food.

## Discussion

We present the case of a 30-year-old woman with anorexia nervosa who developed intermittent fever while transitioning from continuous to intermittent tube feeding, with an increase in protein intake. The fever was diagnosed as being related to DIT and resolved the day after protein intake in the tube feeding decreased.

DIT refers to the production of heat during the digestion and absorption of food in the gastrointestinal tract [1,5]. The body’s energy production varies based on the nutrient types, with reported percentages of approximately 20-30% for protein, 5-10% for carbohydrates, and 0-3% for fat, with protein having the highest percentage [6].

The goal of anorexia nervosa treatment is to improve the nutritional status of the patients. However, achieving expected weight gain can be challenging owing to increased calorie requirements (30-100 kcal/kg/day) for individuals with anorexia nervosa, given their heightened metabolism [7,8]. Studies have consistently noted elevated DIT in individuals with anorexia nervosa [4,7,9].

In our case, a patient with anorexia nervosa experienced increased fever after transitioning from continuous high-protein tube feeding to intermittent administration and an increased dose. Extensive examinations ruled out potential causes, such as aspiration, infection, or drug fever, attributing the elevated temperature to DIT from high protein administration. Some reports have suggested that intermittent administration has a greater effect on muscle protein synthesis than continuous administration, which may be associated with increased body temperature [10]. Indirect calorimetry, which calculates energy expenditure from the concentration and volume of oxygen and carbon dioxide in exhaled air [11], if used, could have provided objective data on DIT in addition to body temperature. Although studies have reported an association between anorexia nervosa and DIT, there are no case reports of a specific increase in body temperature.

## Conclusions

We present a case of anorexia nervosa with intermittent fever while transitioning from continuous to intermittent tube feeding, with an increase in protein intake. Although there are many unknowns regarding the metabolism of patients with anorexia nervosa, high-protein DIT should be considered in patients with fever of unknown origin. Considering DIT may prevent the unnecessary use of antibiotics and ensure that essential medications are continued.
